# Mass loss and nutrient release during the decomposition of sixteen types of plant litter with contrasting quality under three precipitation regimes

**DOI:** 10.1002/ece3.6129

**Published:** 2020-03-12

**Authors:** Ningning Du, Wenrao Li, Liping Qiu, Yanjiang Zhang, Xiaorong Wei, Xingchang Zhang

**Affiliations:** ^1^ College of Natural Resources and Environment Northwest A&F University Yangling China; ^2^ State Key Laboratory of Soil Erosion and Dryland Farming on the Loess Plateau Northwest A&F University Yangling China; ^3^ School of Life Sciences Henan University Kaifeng China; ^4^ University of Chinese Academy of Sciences Beijing China

**Keywords:** functional group, in situ decomposition, leaf chemical properties, nutrient release, precipitation regime

## Abstract

Mass loss and nutrient release during litter decomposition drive biogeochemical cycling in terrestrial ecosystems. However, the relationship between the litter decomposition process and the decomposition stage, precipitation, and litter quality has rarely been addressed, precluding our understanding of how litter decomposition regulates nutrient cycling in various ecosystems and their responses to climate change. In this study, we measured mass loss as well as carbon and nutrient releases during the decomposition of 16 types of leaf litter under three precipitation treatments over 12 months in a common garden experiment (i.e., using standardized soil and climatic conditions). Sixteen types of leaves were divided into three functional groups (evergreen, deciduous, and herbaceous). The objectives were to understand the effects of decomposition stages and precipitation regimes on litter decomposition and to examine the relationship between this effect and chemical properties. The mass loss and release of nitrogen and potassium were significantly higher in the 6‐ to 12‐month stage of decomposition (high temperature and humidity) than in the 0‐ to 6‐month stage. Phosphorus was relatively enriched in evergreen leaves after 6 months of decomposition. The rates of mass loss and nutrient release were significantly greater in herbaceous than in deciduous and evergreen leaves. Increasing precipitation from 400 to 800 mm accelerated mass loss and potassium release but decreased phosphorus release in the 0‐ to 6‐month stage of decomposition. These results highlighted the contribution to and complexity of litter chemical properties in litter decomposition.

## Graphical abstract

We measured mass loss as well as carbon and nutrient releases during decomposition of 16 leaf litters under three precipitation treatments using in situ decomposition experiments. Phosphorus was relatively enriched in evergreen leaves after 6 months of decomposition. The rates of mass loss and nutrient release were significantly greater in herbaceous than in deciduous and evergreen leaves. Increasing precipitation from 400 to 800 mm accelerated mass loss and potassium release but decreased phosphorus release in the 0‐ to 6‐month stage of decomposition.

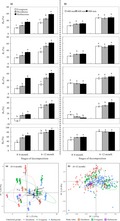

## 1 INTRODUCTION

The decomposition of litter in ecosystems plays an important role in mediating major ecosystem processes (Berg et al., 1993; Salinas et al., 2011; Zhou, Clark, Su, & Xiao, 2015) and sustaining the productivity and functionality of terrestrial ecosystems (Adair et al., 2008). The breakdown of dead plant materials and the release of nutrients drive biogeochemical cycles (Cebrian, 1999; Hirobe, Sabang, Bhatta, & Takeda, 2004; Trogisch, He, Hector, & Scherer‐Lorenzen, 2016) and the availability of nutrients to plants (Swift, Heal, & Anderson, 1979). Litter decomposition is an integral component of the global cycles of nitrogen (N), phosphorus (P), and carbon (C) and the connection between above‐ and belowground biotic processes (Trogisch et al., 2016). Previous estimations have shown that approximately 90% of global terrestrial plant production is transferred into the dead organic matter pool, and the rest is consumed by herbivores (Cebrian, 1999). Among the production transferred into dead organic matter, approximately 76% is lost to the atmosphere (1.9 Mg C ha^−1^ year^−1^) and 24% is redistributed into the soil (Chambers, Schimel, & Nobre, 2001). Therefore, mass loss and nutrient release during the decomposition of litter have important links to ecosystem processes (Berg & McClaugherty, 2013). China's Loess Plateau has semiarid climate conditions (Qiu, Zhang, Cheng, & Yin, 2010). In recent years, vegetation has been restored on the Loess Plateau, and the ecological environment has been improved through a series of measures, such as returning farmland to forests and grasslands and prohibiting logging and grazing. With the development of these projects, the artificial addition of chemical or organic fertilizers has gradually decreased, and plant litter has become the main source of soil organic matter and nutrients in the area. Therefore, the study of the decomposition of plant litter on the Loess Plateau has important theoretical and guiding significance.

Mass loss and nutrient release during decomposition are driven by decomposer interactions with biotic and abiotic factors (Campbell et al., 2016; Cleveland et al., 2014; Lehmann & Kleber, 2015; Tamura, Suseela, Simpson, Powell, & Tharayil, 2017); most of these factors (e.g., soil moisture and temperature, matrix quality, annual evapotranspiration, and soil biological activity) are related to precipitation and temperature (Berg & McClaugherty, 2013; Sanaullah, Rumpel, Charrier, & Chabbi, 2012). In most terrestrial ecosystems, soil moisture, which is largely affected by precipitation, is more important than temperature in influencing litter decomposition (Gray & Fierer, 2012; Hao, Zhao, Zhao, & Wang, 2010). Studies have shown that variation in microclimate at local scales is an important factor for explaining differences in decomposition rates (Bradford, Berg, Maynard, Wieder, & Wood, 2016; Joly et al., 2017). Nevertheless, water availability could become the dominant factor influencing litter decomposition at local scales, particularly in desert or semiarid regions where water is the primary limiting factor (Couteaux, Bottner, & Berg, 1995; Zhang, Hui, Luo, & Zhou, 2008; Zhang, Zhang, & Gao, 2008). Precipitation has a decisive effect on the spatial distribution of vegetation in most areas of the Loess Plateau and is the main factor influencing the growth and development of vegetation on this plateau. A positive correlation between litter decomposition and precipitation has been observed in tropical rain forests with annual precipitation amounts higher than 5,000 mm (southwestern Costa Rica, Wieder, Cleveland, & Townsend, 2009), arid deserts (the northern Chihuahuan Desert, USA, François, Kurupas, & Throop, 2017), and areas with a midcontinental climate (northeastern Kansas, USA, Reed, Blair, Wall, & Seastedta, 2009). Changes in precipitation can influence litter decomposition by directly restricting the physical breakdown of litter and by indirectly affecting the activity of decomposers (Salamanca, Kaneko, & Katagiri, 2003). In addition, litter decomposition studies conducted in Mediterranean systems found a significant reduction in decomposition with reduced water availability (e.g., Almagro, Maestre, Martĺnez‐LÓpez, Valencia, & Rey, 2015; Santonja, Fernandez, Gauquelin, & Baldy, 2015; Santonja et al., 2017). Previous studies have provided an understanding of how precipitation affects mass loss during decomposition, although the effects on nutrient release have been considered less but are urgently needed given their potential to influence the cycling and availability of nutrients in terrestrial ecosystems (Bloor & Bardgett, 2012; Liu, Liu, Guo, Wang, & Yang, 2014) and the coupled cycling of water and nutrients in most ecosystems, particularly in arid and semiarid climates (Suseela, Tharayil, Xing, & Dukes, 2015).

Litter quality has important regulatory effects on decomposition (Maeda, 2016; Vanholme et al., 2012; Zhou et al., 2015). Among the metrics of litter quality, the contents of N and lignin and the ratios of carbon to N (C:N) and lignin:N are considered to be directly related to mass loss and nutrient release during decomposition (Aerts, Van‐Odegom, & Cornelissen, 2012; Berg & McClaugherty, 2011; Li, Han, & Hu, 2008; Melillo, Aber, & Muratore, 1982; Suseela & Tharayil, 2017). For example, Zhang, Hui, et al. (2008), Zhang, Zhang, et al. (2008) found that litter decomposition rates increased with N, P, and K but decreased with C:N, lignin, and lignin:N. Melillo et al. (1982) also showed that litter with high contents of N and P but low C:N ratios decomposed faster than litter with high contents of cellulose, hemicellulose, and lignin. Similar results were reported by Stohlgren (1988a, 1988b). These results, together with many others, indicate that initial concentrations of N, lignin in plant litter, and lignin:N could be good predictors of litter decomposition rates in many ecosystems (Bryant, Holland, Seastedt, & Walker, 1998; Fioretto, Di, Papa, & Fuggi, 2005; Liu, Chen, et al., 2016; Suseela & Tharayil, 2017). Furthermore, the chemical composition of litter during decomposition is affected by precipitation. Hobbie (2000) found that the litter lignin concentration declined with decreasing precipitation in a Hawaiian montane forest. However, to date, whether the regulatory effect of litter quality on mass loss and nutrient release varies with precipitation and decomposition stage has not been examined. There is no quantitative model for the contribution of the chemical properties of litter to mass loss and nutrient release at different stages of decomposition, which hinders the prediction of the effects of litter decomposition on biogeochemical cycles under various global change scenarios.

In this study, we selected leaf litter from 16 plants (four evergreen trees, six deciduous trees, and six herbaceous) to compose a wide range of leaf chemical properties (e.g., N and lignin contents, and C:N and lignin:N ratios), allowing us to examine the regulatory effect of leaf chemical properties on mass loss and nutrient release and whether this regulation varies with precipitation and decomposition stage. We present results regarding mass loss and nutrient release during the decomposition of the leaf litter under three precipitation scenarios to test the following hypotheses: (H1) Mass loss and nutrient release will increase with precipitation, and this response will be consistent across three functional groups of leaves; (H2) there will be a stronger mass loss and nutrient release response to different precipitation regimes in the early compared to the late stage of decomposition; and (H3) there is a strong correlation between the chemical properties of litter, and the contribution of these properties to mass loss and nutrient release will follow different models at different stages of decomposition. The objectives were formulated to understand how precipitation and decomposition stage affect mass loss and nutrient release during decomposition and to relate these processes to leaf chemical properties. Such understanding will provide essential information regarding the relationship of litter decomposition parameters and litter traits as well as precipitation, which will be helpful to support the validation and refinement of terrestrial ecological models.

## 2 MATERIALS AND METHODS

### 2.1 Study site

This study was carried out in the field experimental site of Northwest A&F University. The site is located in Yangling, Shaanxi, China (34°16′18″N, 108°04′59″E, 525 m a.s.l.), with a mean annual precipitation and temperature of 580.5 mm and 15.6°C, respectively, and it is characterized by a warm‐temperate monsoon climate (Zhang et al., 2012). The monthly mean precipitation and air temperature over a period of 53 years are presented in Figure S1. The soil used in this study was collected from farmland (pH = 7.8) that had been abandoned for at least 8 years. The soil is a Typ‐Eum‐Orthic anthrosol, which belongs to the Nitisols according to FAO taxonomy (FAO‐UNESCO, 1988). The concentrations of organic matter, total nitrogen, extractable phosphorus, and potassium were 22.04, 1.63 g/kg, 48, and 490 mg/kg, respectively. The texture of the soil was clay loam, and the gravimetric moisture content at field capacity was 22%. The contents of sand, silt, and clay were 27%, 41%, and 32%, respectively. Given that all the soil used in this study was the same, the effects of the initial soil properties on litter decomposition would be negligible.

### 2.2 Litter material and sampling

To generate a wide span of leaf chemical properties, we selected leaves from 16 species that are widely distributed in Shaanxi Province and vary markedly in their leaf chemical properties. We selected these species according to the results from our previous investigation so that the leaf chemical properties varied significantly. The 16 species belonged to six deciduous trees, four evergreen trees, and six herbaceous. The deciduous trees were *R. pseudoacacia* (*Robinia pseudoacacia*, Leguminosae), *P. ningshanica* (*Populus ningshanica*, Salicaceae), *M. pumila* (*Malus pumila*, Rosaceae), *Q. wutaishanica* (*Quercus wutaishanica*, Fagaceae), *B. platyphylla* (*Betula platyphylla*, Betulaceae), and *S. psammophila* (*Salix psammophila*, Salicaceae). The evergreen trees were *P. orientalis* (*Platycladus orientalis*, Cupressaceae), *C. deodara* (*Cedrus deodara*, Pinaceae), *P. tabuliformis* (*Pinus tabuliformis*, Pinaceae), and *P. serrulata* (*Photinia serrulata*, Rosaceae). The herbaceous species were *S. capillata* (*Stipa capillata*, Gramineae), *G. max* (*Glycine max*, Leguminosae), *O. sativa* (*Oryza sativa*, Gramineae), *Z. mays* (*Zea mays*, Gramineae), *H. annuus* (*Helianthus annuus*, Compositae), and *S. italica* (*Setaria italica*, Gramineae). In August 2014, fresh green leaves were collected before most of the leaves naturally fell and were oven‐dried (40°C) and stored at room temperature. The sampling site and chemical composition of the 16 types of leaves are presented in Table 1.

**Table 1:** The sampling sites and the mean values of the initial chemical properties of each leaf litter used in this study

	Common name	Latin name	Sampling site	C (g/kg)	N (g/kg)	P (g/kg)	K (g/kg)	C:N	Cellulose (g/kg)	Lignin (g/kg)	Lignin:N
Deciduous	Locust	*Robinia pseudoacacia*	Fufeng (34°37′N, 107°45′E)	413.3	25.6	1.5	4.3	16.1	294	191	7.5
	Poplar	*Populus ningshanica*	Yangling (34°16′N, 108°7′E)	411.7	29.0	2.3	9.6	14.2	298	234	8.1
	Apple	*Malus pumila*	Changwu (35°18′N, 107°38′E)	426.3	24.8	1.7	14.7	17.2	211	132	5.3
	Birch	*Betula platyphylla*	Fuxian (36°23′N, 108°42′E)	469.6	18.7	2.4	11.5	25.1	443	154	8.2
	Liaodong oak	*Quercus wutaishanica*	Fuxian (36°23′N, 108°42′E)	438.3	17.8	1.6	7.4	24.6	306	201	11.3
	Salix	*Salix psammophila*	Shenmu (38°58′N, 110°30′E)	445.1	17.8	1.9	12.4	25.0	237	323	18.2
Evergreen	Pine	*Pinus tabuliformis*	Yangling (34°16′N, 108°7′E)	488.0	12.4	1.4	5.7	39.5	286	306	24.8
	Cedar	*Cedrus deodara*	Fufeng (34°37′N, 107°45′E)	447.6	12.6	0.9	4.9	35.5	371	223	17.7
	Arborvitae	*Platycladus orientalis*	Yangling (34°16′N, 108°7′E)	501.0	10.5	1.1	3.2	47.8	420	205	19.6
	Heather	*Photinia serrulata*	Yangling (34°16′N, 108°7′E)	430.4	14.1	1.3	8.5	30.5	294	238	16.9
Herbaceous	Sunflower	*Helianthus annuus*	Hengshan (37°21′N, 110°01′E)	332.1	40.5	3.4	27.5	8.2	191	105	2.6
	Maize	*Zea mays*	Yangling (34°16′N, 108°7′E)	413.0	26.1	2.9	17.3	15.8	271	185	7.1
	Millet	*Setaria italica*	Hengshan (37°21′N, 110°01′E)	370.8	20.8	2.1	17.8	17.8	272	173	8.3
	Stipa	*Stipa capillata*	Guyuan (36º38′N, 106º58′E)	402.4	12.4	1.1	8.6	32.4	319	252	20.3
	Soybean	*Glycine max*	Hengshan (37°21′N, 110°01′E)	388.7	35.2	2.5	8.1	11.0	250	164	4.7
	Rice	*Oryza sativa*	Hengshan (37°21′N, 110°01′E)	385.9	27.0	2.6	14.7	14.3	251	152	5.6

Note

C: carbon; N: nitrogen; P: phosphorus; K: potassium.

As nearly all the leaves fell at the end of the growing season (autumn), our experiment started on 30 November 2014. The litter remaining in each container after 6 and 12 months of decomposition was collected with tweezers and placed in paper bags. For some samples, a very small amount of soil particles was attached to the decomposed litter, and a soft brush was used to remove these attached soil particles very carefully. Therefore, there was very little or no mineral soil attached to the litter, and thus, litter was not analyzed for percentage ash. The litter was oven‐dried at 60°C to a constant weight, weighed to calculate the mass loss during decomposition, and then ground to pass through a 0.25‐mm sieve for chemical analysis.

### 2.3 Experimental setup

In situ litter decomposition experiments were carried out in the field using PVC (polyvinyl chloride) containers (20 cm in diameter and 20 cm in depth), 10 cm of which was buried in the soil. Six grams of leaf litter was placed into the containers and on the surface of the soil. The samples in the containers were divided into two parts by a separator: One side was designed for the 6‐month decomposition, corresponding to an early stage of decomposition, and the other side was designed for the 12‐month decomposition and was used for calculating the results of late‐stage decomposition by a comparison with the results from the 6‐month decomposition. A nylon net (20 cm in diameter with a 2 mm mesh size) was carefully placed on the litter to minimize the loss of litter by wind and other factors. The details of the experimental design are shown in Figure S3. To minimize the effect of spatial variations in light, temperature, and rainfall, a plastic shed was set up at a 3‐m height above the experimental units. The plastic shed was 11 m long and 6 m wide (Figure S3).

**Figure 1:**
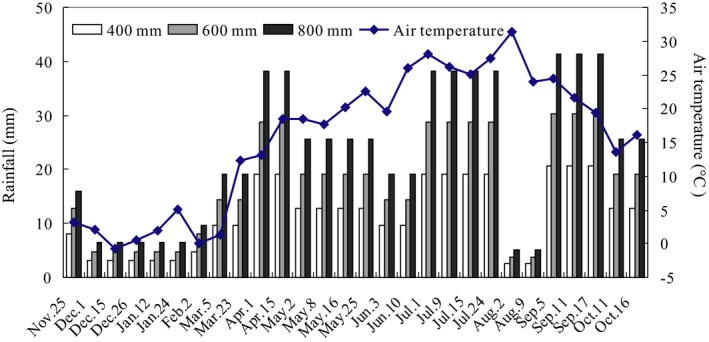
The manipulated rainfall in 400, 600, and 800 mm precipitation treatments and air temperature during the experimental period (November 2014–October 2015)

The experimental design included the decomposition stage, precipitation treatments, and litter types. The decomposition stages included 0–6 and 6–12 months of decomposition. Because all 16 plant species used in this study are widely distributed in Shaanxi Province, which is characterized by a precipitation range of 400–800 mm with a mean precipitation of 600 mm, the designed precipitation treatments in this study were 400, 600, and 800 mm per year, which are capable of capturing the range and average precipitation regimes. Moreover, the average precipitation regime used in this study (600 mm) is similar to the 53‐year mean annual precipitation at the study site (580.5 mm). The precipitation was controlled by an artificial simulation system (Figure S4), and the rainfall amounts in each treatment during the experimental periods are presented in Figure 1. An artificial simulated rainfall system was installed at the top of the shed. The spray range of the water sprinkler was short, the spray was stable, and a 360° full‐circle spray was applied. The water droplets sprayed from the sprinkler were small enough to simulate raindrops (Figure S4). Each type of leaf litter received a combination of the decomposition stage and precipitation treatments with six replicates, resulting in a total of 288 plots.

### 2.4 Chemical analysis

The litter before decomposition was prepared for the analysis of the concentrations of C, N, P, K, lignin, and cellulose. The samples after 6 and 12 months of decomposition were prepared for the analysis of the C, N, P, and K concentrations. The concentration of C in the litter samples was analyzed using the wet combustion method with potassium dichromate and sulfuric acid oxidation for 5 min followed by titration with 0.2 mol/L FeSO_4_ (Nelson & Sommers, 1982). A separate sample was wet‐combusted with H_2_SO_4_‐H_2_O_2_, and a solution was prepared for the measurement of the N, P, and K concentrations (Page, Miller, & Keeney, 1982). The concentration of N in solution was titrated with 0.05 mol/L HCl using an auto Kjeldahl analyzer (FOSS Kjeltec 2300). The concentration of phosphorus in solution was measured by the molybdenum blue colorimetry method. The concentration of potassium in solution was analyzed by the flame emission method (Thermo ICE 3000). The lignin and cellulose concentrations were determined according to the acid detergent lignin method (Vanderbilt, White, Hopkins, & Craig, 2008) with some modifications (He et al., 2016). Briefly, fine litter samples (1.0 g) that were oven‐dried and ground were transferred to digestion tubes and suspended in a solution of H_2_SO_4_ (1.0 mol/L) and cetyltrimethylammonium bromide (CTAB; 20 g/L; 80 ml). The tubes were heated at 169°C for 1 hr. After cooling, the tubes were transferred to a sand core funnel (50 ml, G3 specification) and washed with acetone until the solution obtained through the suction filtration was clean. After oven‐drying at 170°C for 1 hr, the sample and tube were weighed together and were designated as W1. Subsequently, the sample was soaked for more than 3 hr in an H_2_SO_4_ solution (72%), subjected to suction filtration, and washed with acetone, as described above. The sample was then oven‐dried at 170°C for 1 hr. The sample and tube were then weighed together and were designated as W2, which was placed in a muffle furnace (Box Furnace; Lindberg/Blue M) at 550°C for 3 hr and weighed after cooling (designated as W3). The cellulose concentration was determined from the weight loss difference between W1 and W2 divided by the sample weight (1.0 g) and then multiplied by 100, whereas the lignin concentration was determined as the difference between the W2 and W3 measurements. All analyses were conducted in triplicate.

The difference between the initial amounts and those remaining after 6 months was used to calculate total C, N, P, and K release during the 0–6 months of decomposition. The difference between the amounts remaining after 6 and 12 months was used to calculate total C, N, P, and K release during the 6–12 months of decomposition.

### 2.5 Data analyses

In this study, we defined the early and late stages of decomposition as the 0‐ to 6‐month and 6‐ to 12‐month decomposition, respectively. The rate of mass loss (R_M_, %) during litter decomposition in each treatment was calculated as follows:

(1) $$R_{M}=\frac{M_{0}-M_{t}}{M_{0}}\times 100\%$$

where M_0_ and M_t_ are the mass (g) of the litter before and after decomposition, respectively, for each stage.

The release rates of C, N, P, and K (R_C_, R_N_, R_P_, and R_K_, respectively, %) were calculated as follows:

(2) $$R_{C}=\frac{M_{0}\times C_{0}-M_{t}\times C_{t}}{M_{0}\times C_{0}}\times 100\%$$

(3) $$R_{N}=\frac{M_{0}\times N_{0}-M_{t}\times N_{t}}{M_{0}\times N_{0}}\times 100\%$$

(4) $$R_{P}=\frac{M_{0}\times P_{0}-M_{t}\times P_{t}}{M_{0}\times P_{0}}\times 100\%$$

(5) $$R_{K}=\frac{M_{0}\times K_{0}-M_{t}\times K_{t}}{M_{0}\times K_{0}}\times 100\%$$

where C_0_, N_0_, P_0_, and K_0_ are the concentrations (g/kg) of carbon, nitrogen, phosphorous, and potassium, respectively, in the litter samples before decomposition in each stage, and C_t_, N_t_, P_t_, and K_t_ are the concentrations of carbon, nitrogen, phosphorus, and potassium, respectively, in the litter samples after decomposition in each stage.

Multiway analysis of variance (ANOVA) was conducted to examine the direct and interactive effects of decomposition stage (0–6 months vs. 6–12 months), precipitation (400, 600, and 800 mm), and functional group of the litter (grass and crops, deciduous, and evergreen) on the rates of mass loss and carbon and nutrient release (Table 2). In this study, lignin and cellulose were not measured for the samples after 6 and 12 months of decomposition because the amount of residue was not sufficient for the measurement, so the analysis of chemical properties was divided into two decomposition stages. Correlation coefficients among the chemical properties of the respective litter in the 0‐ to 6‐month (Table S2) and 6‐ to 12‐month (Table S3) decomposition stages were also analyzed. Principal component analysis (PCA) was used to identify the relationships between leaf chemical properties of different functional groups (deciduous, evergreen, and herbaceous) in 0‐ to 6‐month and 6‐ to 12‐month stages of decomposition (Figure 4). All the statistical analyses were carried out using SPSS software (version 19.0, IBM). Principal component regression analysis was performed using the R software package v.3.3.2 (R Core Team, 2018).

**Table 2:** Multiway analysis of variance (ANOVA) for the effects of decomposition stages (ST, 0‐ to 6‐month vs. 6‐ to 12‐month stage), precipitation treatments (PP, 400, 600, and 800 mm per year), and functional groups (FG) on the rates of mass loss and nutrients release

	R_M_ (%)		R_C_ (%)		R_N_ (%)		R_P_ (%)		R_K_ (%)	
	*F*	*p*	*F*	*P*	*F*	*p*	*F*	*p*	*F*	*p*
FG	62.1	<.001	61.1	<.001	123.3	<.001	35.1	<.001	91.4	<.001
PP	2.1	.137	3.1	.047	0.9	.422	1.2	.317	6.6	.001
ST	152.9	<.001	245.5	<.001	103.6	<.001	509.8	<.001	561.3	<.001
FG × PP	0.8	.525	0.5	.754	1.3	.262	0.4	.821	0.4	.797
FG × ST	2.1	.130	4.5	.012	5.5	.004	3.7	.025	22.6	<.001
ST × PP	1.5	.229	2.8	.065	1.5	.217	16.9	<.001	12.0	<.001
ST × FG×PP	0.2	.916	0.4	.829	0.6	.694	0.1	.983	1.1	.343
RMSE	1.308		1.306		1.439		1.609		1.108	
*R* ^2^	.348		.440		.422		.540		.585	
*p*	<.001		<.001		<.001		<.001		<.001	
*F*	10.567		6.460		9.734		4.179		3.010	

Note

R_M_: mass loss rate; R_C_: carbon release rate; R_N_: nitrogen release rate; R_P_: phosphorous release rate; R_K_: potassium release rate; RMSE: root mean square error.

## 3 RESULTS

### 3.1 Mass loss and carbon release during decomposition

**Figure 2:**
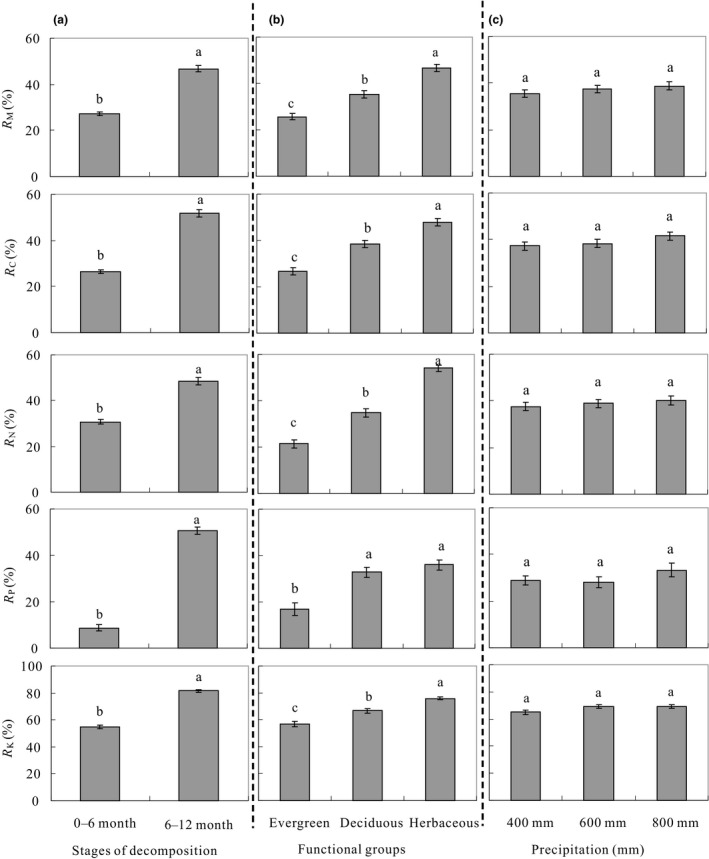
(a) The mass loss rate (R_M_, %) and release rates of carbon (R_C_, %), nitrogen (R_N_, %), phosphorous (R_P_, %), and potassium (R_K_, %) in 0‐ to 6‐month and 6‐ to 12‐month stages of decomposition. Different lowercase letters indicate significant difference between 0‐ to 6‐month and 6‐ to 12‐month stages of decomposition. (b) The mass loss rate (R_M_, %) and release rates of carbon (R_C_, %), nitrogen (R_N_, %), phosphorous (R_P_, %), and potassium (R_K_, %) in different functional groups. Different lowercase letters indicate significant difference in different functional groups. (c) The mass loss rate (R_M_, %) and release rates of carbon (R_C_, %), nitrogen (R_N_, %), phosphorous (R_P_, %), and potassium (R_K_, %) in different precipitation treatments. Different lowercase letters indicate significant difference in different precipitations. Error bars denote two standard errors of the mean

**Figure 3:**
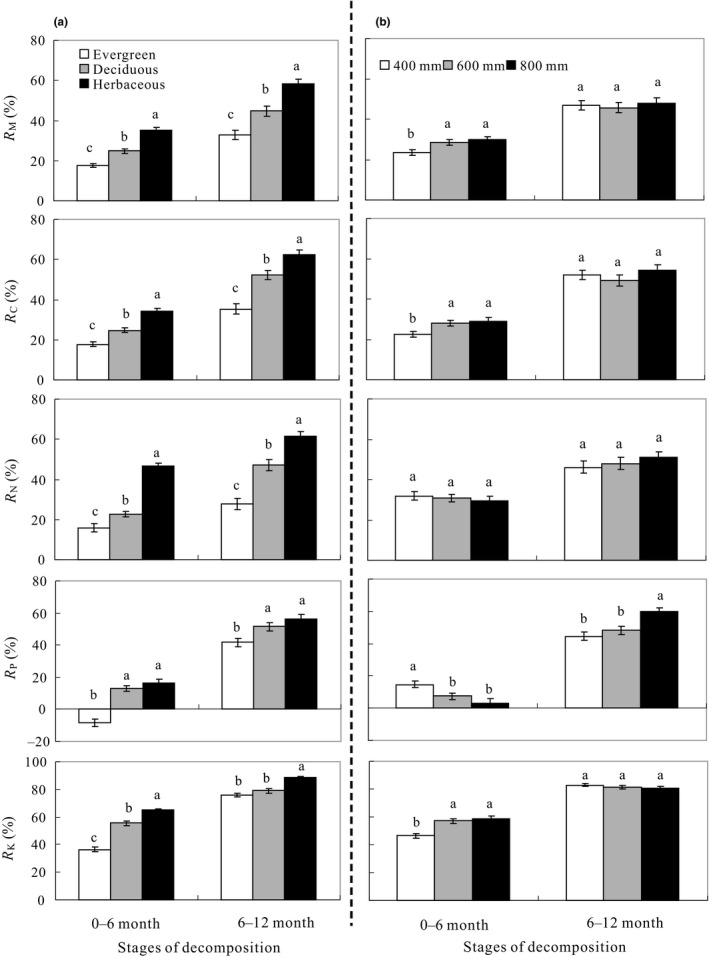
(a) The mass loss rate (R_M_, %) and release rates of carbon (R_C_, %), nitrogen (R_N_, %), phosphorus (R_P_, %), and potassium (R_K_, %) in 0‐ to 6‐month and 6‐ to 12‐month stages of decomposition in different functional groups. Different lowercase letters indicate significant difference among functional groups in same decomposition stage. (b) The mass loss rate (R_M_, %) and release rates of carbon (R_C_, %), nitrogen (R_N_, %), phosphorous (R_P_, %), and potassium (R_K_, %) in 0‐ to 6‐month and 6‐ to 12‐month stages of decomposition under 400, 600, and 800 mm precipitation treatments. Different lowercase letters indicate significant difference among precipitation treatments in same decomposition stage. Error bars denote two standard errors of the mean

As expected, the rate of mass loss (R_M_) was significantly higher in the 6‐ to 12‐month stage of decomposition (46.69 ± 1.5%, mean ± *SE*) than that in the 0‐ to 6‐month stage of decomposition (27.25 ± 0.8%) when averaged across the 16 leaf types and three precipitation treatments (*p* < .001, Figure 2a). Furthermore, the effects of precipitation on the R_M_ varied with the decomposition stage (Figure 3b). The average R_M_ across the 16 species was significantly higher in the 600 and 800 mm treatments (28.29 ± 1.4 and 29.94 ± 1.5%, respectively) than in the 400 mm treatment (23.43 ± 1.3%) (*p* < .05) in the 0‐ to 6‐month stage but was not affected by the precipitation treatments in the 6‐ to 12‐month stage (46.79 ± 2.3, 45.72 ± 2.6, and 47.60 ± 2.8% for the 400, 600, and 800 mm treatments, respectively).

The R_M_ was significantly higher in the litter from the herbaceous (46.59 ± 1.5%) but was significantly lower in the litter from the evergreen trees (25.68 ± 1.4%) compared with the litter from the deciduous trees (35.22 ± 1.6%) (*p* < .001) when averaged across the two decomposition stages and precipitation treatments (Figure 2b). Although there was great variation in the R_M_ among the litter from the 16 species (Table [Link], [Link]), the effects of the functional group were consistent across the decomposition stage, with a significantly higher R_M_ for the herbaceous but a lower R_M_ for the evergreen trees within each of the combinations (Figure 3a). Therefore, the functional groups dominated the mass loss during the decomposition of the leaf litter regardless of the decomposition stage and precipitation treatments in this study.

The release rate of carbon (R_C_) during litter decomposition was significantly and positively correlated with the R_M_ (Figure S2) and therefore showed a similar response pattern to the decomposition stage, precipitation treatment, and functional groups in this study (Table 2).

### 3.2 Nutrient release during decomposition

The release rate of N during decomposition (R_N_) was 108% higher in the 6‐ to 12‐month stage than in the 0‐ to 6‐month stage of decomposition (*p* < .001) and varied with the functional groups of the leaves (*p* < .001) (Figures 2a and 3a) The R_N_ was significantly higher in the leaf litter from the herbaceous (53.94 ± 1.3%) but lower in the leaf litter from the evergreen trees (21.50 ± 1.8%) compared with the litter from the deciduous trees (34.84 ± 1.8%) when averaged across the two stages and three precipitation treatments (*p* < .001, Figure 2b). The R_N_ in the 6‐ to 12‐month stage of decomposition was 37, 156, and 191% higher than in the 0‐ to 6‐month stage for the herbaceous, evergreen, and deciduous trees, respectively, when averaged across the three precipitation treatments (Figure 3a).

The R_N_ was not affected by the precipitation treatment in the 0‐ to 6‐month and 6‐ to 12‐month stages of decomposition (Figure 3b). When tested within each functional group, no significant differences in R_N_ in response to precipitation were observed. Therefore, the R_N_ was less dependent on precipitation than on the functional group and stage of decomposition in this study.

The release rates of P and K during decomposition (R_P_ and R_K_) were significantly affected by the functional group of the leaves and the stage of decomposition (Table 2). When averaged across the two stages of decomposition, the R_P_ and R_K_ were significantly higher for the herbaceous (35.94 ± 2.2 and 75.94 ± 1.1%) than for the evergreen trees (16.94 ± 2.3 and 56.86 ± 2.1%) and deciduous trees (32.86 ± 2.1 and 66.59 ± 1.4%) (Figure 2b). When averaged across the three functional groups of leaves, the R_P_ and R_K_ were 460 and 78% higher, respectively, in the 6‐ to 12‐month stage than in the 0‐ to 6‐month stage of decomposition (*p* < .001) (Figure 3a).

In this study, the R_P_ and R_K_ were not affected by the precipitation treatment when averaged across the two stages of decomposition (Figure 2c). In the 0‐ to 6‐month stage of decomposition, the R_P_ was significantly higher in the 400 mm precipitation treatment (14.71 ± 2.0%) but lower in the 600 mm (7.31 ± 1.9%) and 800 mm (3.05 ± 3.0%) precipitation treatments. In the 6‐ to 12‐month stage of decomposition, the R_P_ was significantly higher in the 800 mm precipitation treatment (*p* < .05), which was the opposite of the 0‐ to 6‐month stage of decomposition (Figure 3b). The R_K_ increased significantly with precipitation (*p* < .001) in the 0‐ to 6‐month stage of decomposition but was not altered by precipitation (*p* > .05) in the 6‐ to 12‐month stage of decomposition (Figure 3b).

### 3.3 The dependence of mass loss and nutrient release on litter chemical properties

**Figure 4:**
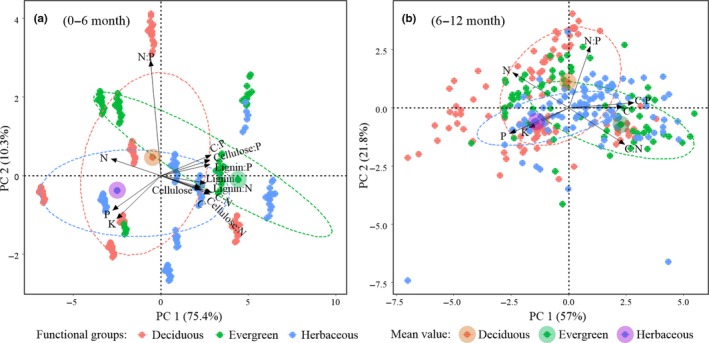
Principal component analysis used to identify the relationships between leaf chemical properties of different functional groups (deciduous, evergreen, and herbaceous) in 0‐ to 6‐month (a) and 6‐ to 12‐month (b) stages of decomposition. Loadings included are displayed as C, N, P, K, C:N, N:P, C:P, cellulose, lignin, cellulose:N, lignin:N, cellulose:P, and lignin:P. C: carbon; N: nitrogen; P: phosphorus; and K: potassium. Principal components 1 and 2 (shown) best described the relationship between leaf chemical properties

There are many leaf chemical properties that affect mass loss and nutrient release. To investigate the correlation between these chemical properties, we performed a whole correlation analysis in the 0‐ to 6‐month (Table S2) and 6‐ to 12‐month (Table S3) stages of decomposition. The results indicate that in this study, there was a significant correlation between all leaf chemistry properties (except for some N:P ratios). Principal component analysis (PCA) was used to identify the relationships between leaf chemical properties of different functional groups (deciduous, evergreen, and herbaceous) in the 0‐ to 6‐month and 6‐ to 12‐month stages of decomposition. Principal components 1 and 2 (shown) best described the relationship between leaf chemical properties: 0–6 and 6–12 months explained 85.7% and 78.8%, respectively (Figure 4). We found that each functional group was evenly distributed in the graph, and different precipitation regimes were also evenly distributed in the graph (not shown). We also found a significant correlation among chemical properties, and the distribution of the chemical properties in the graph did not change much between 0–6 months and 6–12 months (Figure 4). These results indicated that as the decomposition time increased, the same release trend occurred among the compounds.

When looking at the mean value of the three functional groups in Figure 4, we found that N, P, and K can be used as representative chemical properties of herbaceous; N:P can be used as a representative chemical property of deciduous trees; and C, C:N, C:P, and difficult‐to‐decompose substances and their stoichiometric ratios (e.g., cellulose, lignin, cellulose:N, cellulose:P, lignin:N, and lignin:P) can be used as representative chemical properties of evergreen trees. It is interesting that the later stage of decomposition had basically the same response pattern as the early stage of decomposition. Although we did not measure cellulose and lignin in the 6‐ to 12‐month stage, according to the distribution of the characteristics in the figure and the correlation among the characteristics (Tables S2 and S3), we concluded that lignin and cellulose are the representative properties of evergreen trees. Therefore, the chemical properties of the litter itself did not change during decomposition.

## 4 DISCUSSION

Our results showed that mass loss and C and K release were significantly greater in the higher precipitation treatment than in the lower precipitation treatment at the early decomposition stage but were minimally affected by precipitation at the late stage of decomposition. Furthermore, the mass loss and nutrient release were regulated by the litter chemical properties, and the contribution of these chemical properties would have different models at different stages of decomposition. These results partially support our hypotheses and indicate that the time of decomposition and climate together mediate litter decomposition and nutrient cycling.

### 4.1 Mass loss and carbon release during decomposition

In our study, the mass loss rate was higher in the 6‐ to 12‐month (46.69%) than in the 0‐ to 6‐month (27.25%) decomposition stage. This trend was not in agreement with the exponential function of mass loss, where the mass rate decreases with time of decomposition (Corrigan & Oelbermann, 2010; Wu, Li, & Wan, 2013). Previous studies have shown that Creosote bush (*Larrea tridentata*) fine litter mass loss over a 3‐month period accounted for approximately 20% of the original mass (Moorhead & Reynolds, 1989), and litter mass loss after 21 days of incubation in the laboratory was extremely low (<3%) (Gomes, Ferreira, Tonin, Adriana, & José, 2017). The results from Gomes, Medeiros, and Gonçalves (2016) showed 17.5% mass loss for *Maprounea guianensis* litter after 10 days of incubation in laboratory microcosms. Additionally, leaching loss of mass dominated the decomposition in the early stage (Berg & McClaugherty, 2013), while the accumulation of lignin and cellulose and other refractory substances inhibited decomposition at the late stage (Voříšková & Baldrian, 2013). Regarding long‐term decomposition, it has been shown that mass loss in a Mediterranean shrubland in southern France varied between 18.6% (*Ulex*) and 36.5% (*Cistus*) after 1 year and between 27.0% (*Ulex*) and 53.0% (*Quercus*) after 2 years (Santonja et al., 2019). However, it should be noted that our study covered only the early stage of decomposition according to the definition used, and the average temperature (5.2°C) and precipitation (11.8 mm) of the 0‐ to 6‐month stage of decomposition were significantly lower than the average temperature (22.5°C) and precipitation (21.1 mm) of the 6‐ to 12‐month stage of decomposition (Figure 1). A low‐temperature and low‐humidity environment will reduce decomposition (Liu, Chen, et al., 2016; Sardans, Rivas‐Ubach, & Penuelas, 2012; Wang, Li, & Bai, 2000); thus, the decomposition rate in the 0‐ to 6‐month stage was less than that in the 6‐ to 12‐month stage. Therefore, the effects of litter decomposition remain uncertain, mainly due to differences in experimental setups (mass loss classes vs. specific time span) and ecosystem types (forests vs. shrubland).

Our results showed that the R_M_ followed the order of herbaceous > deciduous leaves > evergreen leaves, which is in agreement with previous observations (Cornwell et al., 2008; Gholz, Wedin, Smitherman, Harmon, & Parton, 2000; Wu et al., 2013). For example, Cornwell et al. (2008) found that the decomposition of litter from deciduous species was 60% faster than that from evergreen litter. Similarly, Wu et al. (2013) reported that in a 2‐year in situ decomposition experiment, the mass loss after 336 and 701 days was greater for deciduous leaves (ca. 30% and 40%, respectively) than for evergreen leaves (ca. 15% and 25%, respectively) in Chinese temperate forests. The higher R_M_ of the herbaceous and deciduous leaves than that of the evergreen leaves was mainly due to the lower contents of lignin and cellulose and lower C:N and C:lignin ratios (Wu et al., 2013, Table 1). Gomes et al. (2016) found 17.5% mass loss for *M. guianensis* litter after 10 days of incubation in laboratory microcosms. Moretti, Gonçalves, Ligeiro, and Callisto (2007), Alvim, Medeiros, Rezende, and Gonçalves (2015), and Medeiros et al. (2015) found 5%–15% leaf mass loss for several Cerrado plant species (herbaceous) after 1 week of field incubation and a maximum 20% mass loss after 30 days. Hättenschwiler and Jørgensen (2010) found that at a forest experimental site (consisting of approximately 140 evergreen species) near Sinnamary, French Guiana, after approximately 7 months of field treatment, the average remaining litter mass changed between 25.2% and 71.3%, which is higher than our study for the mass loss during the early stage of decomposition. In this study, we found that the R_M_ and litter C were significantly negatively correlated (Figure S5), which can also explain the changes in the R_M_ between various functional groups of leaves.

### 4.2 Nutrient release during decomposition

We showed that the release rates of nutrients were greater in the 6‐ to 12‐month stage than in the 0‐ to 6‐month stage of decomposition, which was consistent with the effects of stage on the R_M_ and R_C_ (Figure 2a). These results suggest that the release of nutrients is coupled with mass loss during leaf decomposition. We observed a relative enrichment of P (negative R_P_) in the evergreen leaves at the 0‐ to 6‐month stage of decomposition, probably because the mass loss was faster than the P release and leaching from the litter. An alternative explanation is the immobilization of P in microbial biomass at the early stage of decomposition because most decomposition is P‐limited (Liu, Liu, et al., 2016; Liu, Chen, et al., 2016). In this study, among the 16 leaf types, the N, P, and K contents of the evergreen species were significantly lower than those of the deciduous and herbaceous species, while the C, C:N, cellulose, lignin, and lignin:N were significantly higher (Table 1). Therefore, the decomposition of these leaves was limited by the availability of P, while the acquisition of P by microorganisms in soils may result in the relative enrichment of P in litter. This explanation is supported by observations that the P concentration increased gradually during the decomposition of *Malus domestica* leaf litter and was positively correlated with mass loss (Han et al., 2011). In this study, when averaged across all the leaves, the rate of P release in the 6‐ to 12‐month stage (50.74 ± 1.6%) was almost six times the rate in the 0‐ to 6‐month stage of decomposition (8.80 ± 1.4%), probably because P is mainly distributed in refractory substances and combines with lignin and cellulose, which decompose during the late stage of decomposition (Suzuki, Makino, & Mae, 2001).

Our results further showed that significantly higher nutrient release occurred from the herbaceous and deciduous leaves than from the evergreen leaves (*p* < .001, Figure 2b), which is consistent with the patterns of influence of functional groups on the R_M_ (Figure 2b). These results were primarily due to variations in leaf quality and thus decomposition processes, as discussed above. This explanation was supported by previous observations in an alpine forest–tundra ecotone on the eastern Tibetan Plateau, where the nutrient loss from deciduous leaves (ca. 60%) was twice that from evergreen leaves (ca. 30%) after 360 days of decomposition (Liu, Chen, et al., 2016). Pérezsuárez, Arredondomoreno, Hubersannwald, and Vargashernández (2009) also found that after 4 and 7 months of decomposition, N loss from deciduous leaves (ca. 11% and 55%) was higher than that from evergreen leaves (ca.‐25% and 50%) in central‐northwest Mexico. Our results therefore provide further evidence that nutrient cycling is faster and that the soil nutrient content is higher in deciduous forests than in evergreen forests (Arunachalam & Singh, 2002; Nsabimana, Haynes, & Wallis, 2004).

### 4.3 The effects of precipitation on mass loss and nutrients during decomposition

We demonstrated that the effects of precipitation on mass loss varied with decomposition stage, with an accelerated R_M_ during the 0‐ to 6‐month stage due to increased precipitation but an unchanged R_M_ during the 6‐ to 12‐month stage. This is probably because the decomposition of water‐soluble substances and carbohydrates (decomposition mainly in the early stage) is sensitive to precipitation, while the decomposition of lignin, cellulose, and refractory substances (decomposition mainly in the late stage) is relatively stable with precipitation (Wang et al., 2017). Austin and Vitousek (2000) showed that the mass loss of all common litter increases with increasing precipitation in Hawaii, USA. Our results are in agreement with previous observations in tropical (Wieder et al., 2009), temperate (Reed et al., 2009; Song et al., 2009), and arid and semiarid (François et al., 2017; Hao et al., 2010) climates and are in line with the theory that the effects of rainwater on the mass loss of leaf litter are due to the leaching of labile compounds (Halima, Biyanzi, & Ibrahima, 2008; Lockaby, Wheat, & Clawson, 1996; Salamanca et al., 2003). Therefore, changes in the labile fraction of litter during decomposition merit consideration in investigating litter mass loss under various climate conditions.

Our results showed that the R_N_ was not affected by precipitation, suggesting the stable release of litter N with precipitation. This observation contrasts with previous findings that precipitation and thus soil moisture drive N cycling during the decomposition of various types of leaf litter (Burke, Lauenroth, & Parton, 1997) and that the interactions of soil water and N availability regulate litter decomposition (Zhang, Hui, et al., 2008; Zhang, Zhang, et al., 2008). Austin and Vitousek (2000) showed that the initial litter mass in Hawaii, USA, changed significantly with the amount of precipitation, ranging from 500 to 5,500 mm mean annual precipitation, after 2 years of decomposition, and the nitrogen content increased with the increase in precipitation. Similarly, Santonja et al. (2019) indicated that experimental reduction in precipitation will have a negative effect on C and N release after 2 years rather than after 1 year of decomposition. Therefore, the lack of a response of the R_N_ to precipitation in this study might be due to the relatively narrow precipitation regimes (400–800 mm) and shorter decomposition time.

Our results showed that the R_P_ and R_K_ were mostly affected by precipitation in the 0‐ to 6‐month stage of decomposition (Figure 3b). The decreased R_P_ in the 0‐ to 6‐month stage of decomposition in response to precipitation might be due to the limiting effect of P on decomposition. As discussed earlier, P may be transported from soil to litter by microorganisms during the early stage of decomposition, and this process will result in the relative enrichment of P in some litter can and be accelerated by increased precipitation. Austin and Vitousek (2000) also pointed out that immobilization of litter occurred at all locations in a wetland for up to 6 months, and the P loss will be reduced. However, in our study, during the 6‐ to 12‐month stage of decomposition, R_P_ increased significantly with increasing precipitation, indicating that phosphorus fixation only occurred in the first half of the year. The increased R_K_ in the 0‐ to 6‐month stage with precipitation might be ascribed to enhanced decomposition with higher precipitation compared to the lower precipitation treatment. This response pattern was similar to that of mass loss (thus C release), probably because K mainly exists in the cytosol, chloroplasts, and glycophytic tissues (Petra, 2012), which decompose. In this study, C release was significantly positively correlated with K release (Figure S2). Our results of the effects of precipitation on K release are consistent with previous results, which showed that up to 33% of K from leaf litter is lost due to leaching (Gessner & Schwoerbel, 1989) because K is highly mobile and not structurally bound (Mahmood et al., 2009).

Changes in precipitation have indirect effects on mass loss and nutrient release and leaching during litter decomposition by influencing soil moisture (Adair et al., 2008; Suseela, Tharayil, Xing, & Jeffrey, 2013). High precipitation results in high soil moisture and thus high activities of soil microbes and litter decomposition (Allison et al., 2013). In this study, we used a uniform decomposition medium, so it is considered that when the precipitation increases, the soil moisture content will be increased, so the soil moisture during the 12‐month decomposition was not measured. We recommend that soil moisture measurements be included in future research.

### 4.4 The dependence of mass loss and nutrient release on litter chemical properties

The measurement of the initial chemical metrics (C, N, P, and K, and cellulose and lignin contents) enabled us to examine the relationships of these factors with the mass loss and nutrient release during the 0‐ to 6‐month and 6‐ to 12‐month decomposition stages. We demonstrated that leaf chemical properties have important regulations on mass loss and nutrient release during decomposition, which is consistent with previous findings in various ecosystems and litter (Austin & Vitousek, 2000; Ding et al., 2012; Gibson, 2012). The previously reported variation was due to carbohydrate decomposition at the early stage and lignin and cellulose and other refractory substance decomposition at the late stage (Corrigan & Oelbermann, 2010; Lambers, Chapin‐Iii, & Pons, 2008), indicating that the early mass loss was greater than the late mass loss. However, in this study, most litter decomposition mainly occurred at the late stage, mainly due to the higher temperature in this stage than in the early stage, which provides an explanation for the similar determination of leaf mass loss and the release of most nutrients. Studies have also shown that an increase in the N content after 2 months of decomposition in an Alaskan boreal forest accelerated total mass loss and cellulose loss (Talbot & Treseder, 2012). The decomposition of lignin and cellulose and other refractory substances is more dependent on the availability of N than on that of carbohydrates (Lambers et al., 2008; Song et al., 2009; Zhang, Hui, et al., 2008; Zhang, Zhang, et al., 2008). For example, Hobbie (2000) found that the N supply enhanced the mass loss of litter and the decomposition of lignin in a Hawaiian montane forest. Similar to this conclusion, in this study the N content of herbaceous was significantly higher than that of deciduous and evergreen trees, so the mass loss of herbaceous was significantly higher than that of other functional groups. Generally, the rates of mass loss and nutrient release during decomposition decreased with higher initial C, cellulose, and C:N, C:P, N:P, and lignin:N ratios in the leaf litter, and there was no fixed pattern of the effects of N, P, and K. Therefore, lower C, cellulose, and lignin enhanced mass loss and the corresponding nutrient release during decomposition (Gautam, Lee, Song, Lee, & Bong, 2016; Song, Zhou, Gu, & Qi, 2015; Vestgarden, 2001).

Although the mass loss of the three functional groups increased during the 6‐ to 12‐month stage of decomposition, the mass loss of the herbaceous was still significantly higher than that of deciduous and evergreen trees. We found that as the decomposition time increased, the same release trend was observed among the functional group compounds, so the R_M_, R_C_, R_N_, R_P_, and R_K_ were basically the same in the 0‐ to 6‐month and 6‐ to 12‐month stages of decomposition. Therefore, we found that within the range of small precipitation gradients, the mass loss and nutrient release of leaf litter in each functional group did differ greatly and were determined by the chemical characteristics of the litter itself. We speculate that the same conclusion will be obtained over a range of large precipitation gradients and longer decomposition times, and of course, we look forward to further demonstration by researchers in the future.

## 5 CONCLUSION

In this study, a common garden experiment was conducted to measure mass loss and carbon and nutrient release during the decomposition of 16 types of leaf litter in three precipitation treatments. The mass loss and nutrient release were greater in the 6‐ to 12‐month stage than in the 0‐ to 6‐month stage of decomposition and were greater for the herbaceous and deciduous leaves than for the evergreen leaves. Phosphorus was relatively enriched in the evergreen leaves at the early stage of decomposition (0–6 months). Increased precipitation (from 400 to 800 mm) accelerated mass loss and potassium release but decreased phosphorus release at the early stage of decomposition. However, precipitation had less effect on mass loss and nutrient release at the 6‐ to 12‐month stage of decomposition. Furthermore, the results indicate that in this study, there was a significant correlation between all leaf chemistry properties (except for some N:P ratios), and mass loss and nutrient release were significantly correlated with leaf chemical metrics. Within the small range of the precipitation regime, with the extension of the decomposition time, the relationship between the chemical properties of the litter, the mass loss, and nutrient release of leaf litter for each functional group did not differ greatly, and all were determined by their own chemical properties.

In this study, we investigated the mass loss and nutrient release during litter decomposition with respect to decomposition stages and precipitation regimes under controlled conditions. Under field conditions, litter contains fractions of contrasting initial compositions due to the mixture of multiple species and plant parts. Furthermore, litter contains a mix of residual compositions due to multiple cohorts and a mix of litter decomposition stages. Therefore, the mixing effects of various species and various decomposition stages should be further studied to obtain a general pattern closer to field conditions.

## CONFLICT OF INTEREST

None declared.

## AUTHOR CONTRIBUTIONS

Qiu LP, Wei XR, and Du NN planned and designed the research. Du NN and Zhang YJ performed experiments and conducted fieldwork. Du NN analyzed the data. All authors discussed the results and wrote the manuscript.

## Supporting information

FigS1

FigS2

FigS3

FigS4

FigS5

TableS1A

TableS1b

TableS2

TableS3

FigS1‐S5_caption

## Data Availability

Available on Dryad after manuscript acceptance. https://doi.org/10.5061/dryad.8pk0p2njj.
