# Supplementary figures and images for: Mass loss and nutrient release during the decomposition of sixteen types of plant litter with contrasting quality under three precipitation regimes
Source: Ecol Evol. 2020 Mar 12;10(7):3367–82. doi: 10.1002/ece3.6129 (PMC7141022; doi:10.1002/ece3.6129)

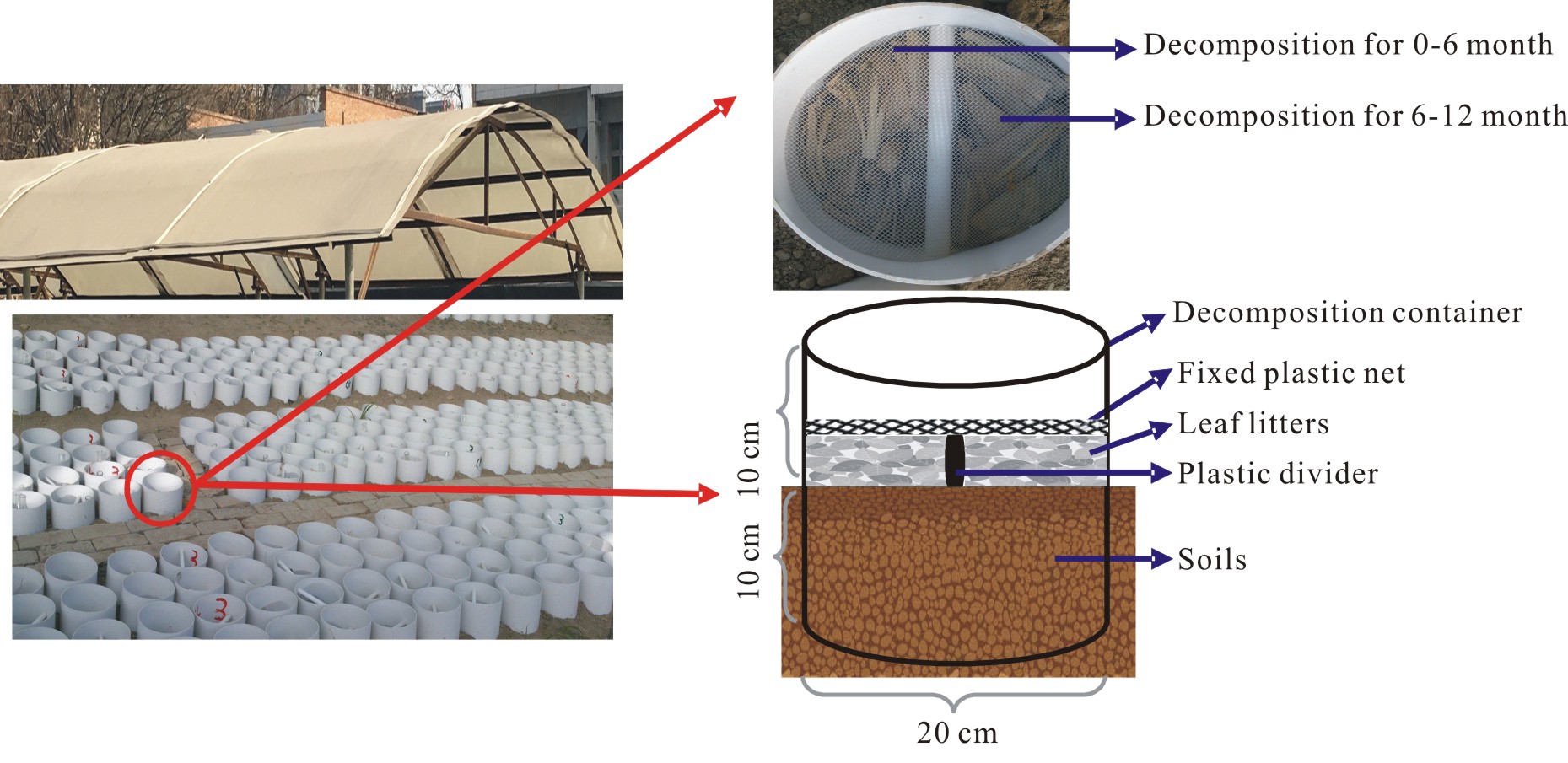

Supplement: Supplementary file 3 — FigS3 [file ECE3-10-3367-s003.jpg]
