# Supplementary material for: Mass loss and nutrient release during the decomposition of sixteen types of plant litter with contrasting quality under three precipitation regimes
Source: Ecol Evol. 2020 Mar 12;10(7):3367–82. doi: 10.1002/ece3.6129 (PMC7141022; doi:10.1002/ece3.6129)
Supplement: Supplementary file 10 — FigS1‐S5_caption [file ECE3-10-3367-s010.docx]

**Figure S1** Monthly mean air temperature and precipitation from 1961-2014 at the study site. Error bars denote two standard errors of the mean.

**Figure S2** Relationship of the mass loss rate (RM,%) to the carbon release rate (RC,%) (left) and the carbon release rate to the potassium release rate (RK,%) (right) during the decomposition of leaf litter.

**Figure S3** Experimental settlements and decomposition container used in this study.

**Figure S4** Artificial simulated rainfall system used in this study.

**Figure S5** The relationships of mass loss rate (RM,%) and release rates of carbon (RC,%), nitrogen (RN,%), phosphorous (RP,%) and potassium (RK,%) versus initial chemical characteristics (C, N, P, K, C:N, N:P, C:P) in litters during 0-6 and 6-12 month stages of decomposition.
